# Pure Air

**Published:** 1858-07

**Authors:** 


### PURE AIR.

THE disagreeable odor of brimstone matches has caused a very general introduction of those made out of *phosphorus*. In the German factories, girls are mostly employed. The fumes of the phosphorus engender a disease of the upper and lower jaw-bones; and in cases where death does not result, these bones are often destroyed. No doubt, the heart of many a reader is moved with pity for these poor girls, who, for the sake of a few cents a day, expose themselves to a living death.

But a fatal disease attacked some of our countrymen, after remaining forty-eight hours at the National Hotel last year. These facts show that impure air may have a more speedy and fatal effect on the human system than arsenic, and should stimulate to a ceaseless vigilance in securing for ourselves and our families, the breathing of a pure atmosphere day and night.

